# Current Advance, Challenges and Future Perspectives of Conduction System Pacing

**DOI:** 10.31083/j.rcm2512438

**Published:** 2024-12-16

**Authors:** Tong-yu Wang, Pei-pei Ma, Yi-heng Yang, Yun-long Xia, Zhao-meng Jing, Zhuang-chuan She, Ying-xue Dong

**Affiliations:** ^1^Department of Cardiology, The First Affiliated Hospital of Dalian Medical University, 116014 Dalian, Liaoning, China

**Keywords:** conduction system pacing, heart failure, cardiac resynchronization therapy, His bundle pacing, left bundle branch pacing

## Abstract

Existing techniques for pacing the right ventricle and providing cardiac resynchronization therapy through biventricular pacing are not effective in restoring damage to the conduction system. Therefore, the need for new pacing modalities and techniques with more sensible designs and algorithms is justified. Although the benefits of conduction system pacing (CSP), which mainly include His bundle pacing (HBP) and left bundle branch area pacing (LBBAP), are evident in patients who require conduction system recuperation, the critical criteria for left CSP remain unclear, and the roles of different pacing modalities of CSP for cardiac resynchronization are not definite. In this review, we aimed to highlight the advantages of different CSP options, current advancement in the surgical devices, and future directions.

## 1. Introduction 

Long-term right ventricular pacing (RVP) has been commonly used as a regular therapy; however, it can lead to electric anomalies such as intra- and inter-ventricular asynchrony or even atrioventricular asynchrony [1]. Furthermore, 15–27% of patients developed heart failure (HF) with left ventricular (LV) electric asynchrony [2].

These elements have led to the suggestion that cardiac resynchronization therapy (CRT) should be used to improve left ventricular ejection fraction (LVEF) and ventricular synchrony. As the standard pacing modality for CRT in patients with HF that require correction of left bundle branch block (LBBB) and atrioventricular block [3]. Biventricular pacing (BiVP) may decrease the rates of mortality and HF and improve remodeling [4]. However, the activation sequence between the LV epicardium and right ventricular (RV) endocardium in BiVP is different from the intrinsic physiological sequence, and approximately 30% patients show no response to CRT. In addition, non-physiological cardiac activation may result in ventricular arrhythmias [5].

If electrical asynchrony is the cause of HF in certain individuals, rebuilding the physiological electrical conductivity is the most logical course of action. The sole method available for capturing the cardiac conduction system is conduction system pacing (CSP), including His bundle pacing (HBP) and left bundle branch area pacing (LBBAP) [6]. The resynchronization improvement is more obvious in HBP than BiVP [7]; however, the drawbacks of HBP, which include a higher pacing threshold and practical challenges, prevent its widespread application [8, 9]. By directly catching the left ventricular branch and avoiding the His bundle, left bundle branch pacing (LBBP) helps to overcome these restrictions [10]. According to the 2023 Heart Rhythm Society’s guidelines, patients with a LVEF of 35–50% are recommended to undergo CSP (IIa) [2]. Although there is mounting evidence to support the benefits of CSP, certain issues, such as the criteria for conduction system capture and the choice of a suitable population, facing this novel modality have likely hindered the widespread use of this pacing method. In this review, we will focus on the clinical implications, limitations, and some unresolved aspects of CSP.

## 2. Cardiac Anatomy Related to CSP

**Fig. 1.**
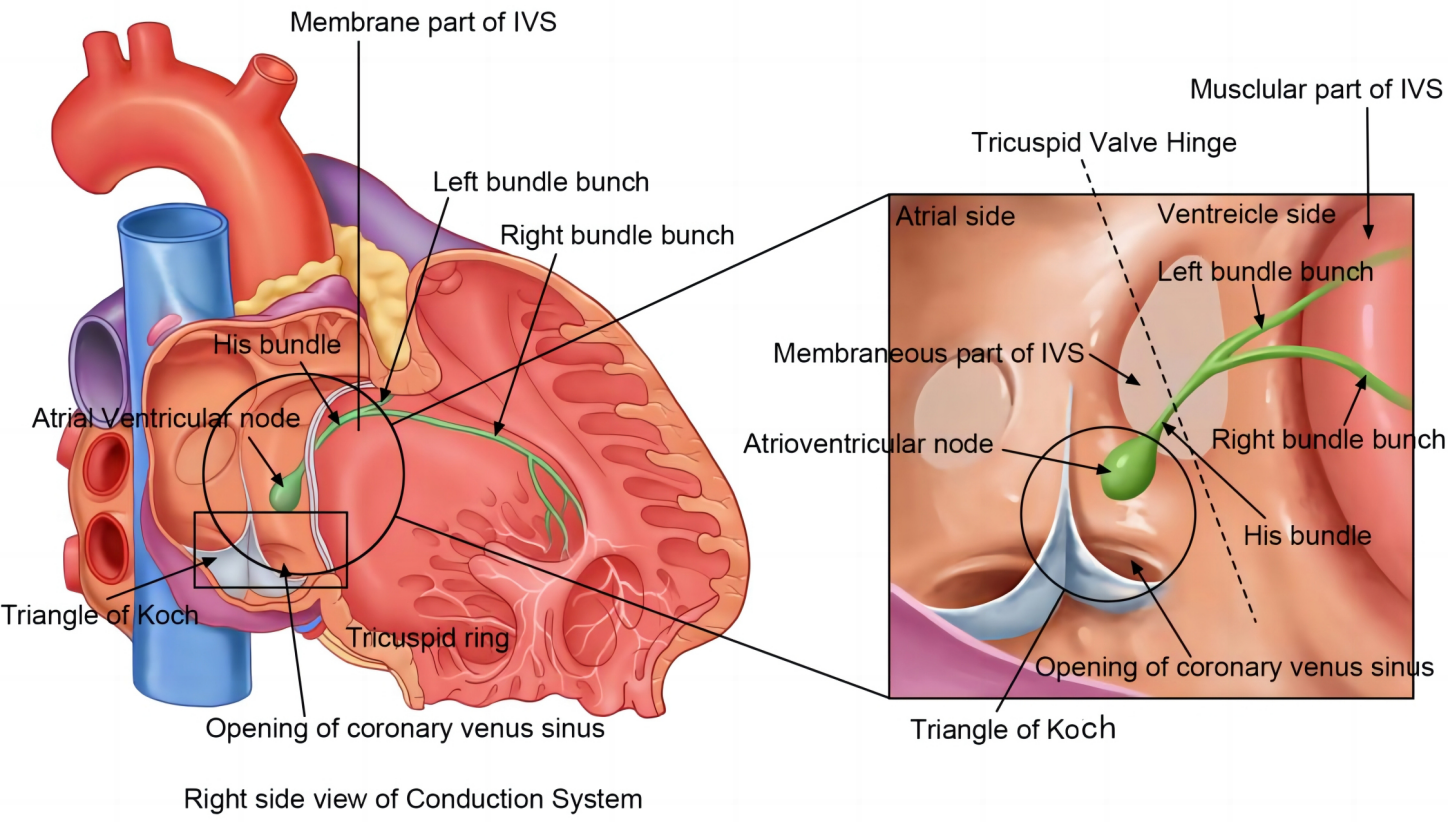
**General anatomy of cardiac conduction system**. An overview of the structure of the cardiac conduction system (right). The atrioventricular node is located deep in the Triangle of Koch. It stretches towards the His bundle, which penetrates the membranous part of the inter-ventricular septum (IVS) before reaching its muscular part and branches into the left bundle branch and right bundle branch. The tricuspid valve ring is hinged at the membranous part of the IVS, where it is divided into the ventricular and atrial parts.

To increase the success of the CSP operation, a thorough understanding of the anatomy and physiology of the cardiac conduction system is essential. The specialized atrioventricular (AV) conduction pathway comprises the compact AV node (AVN), His bundle, right and left bundle branches, and the Purkinje network (shown in Fig. 1) [11].

### 2.1 AVN 

A mass of tiny muscle fibers loosely distributed in an interlacing pattern makes up the AVN. It is situated adjacent to the Triangle of Koch and in the posterior wall of the right atrium, directly beyond the tricuspid valve [12]. A vital component of the atrial musculature is the node. It stops the atria and ventricles from contracting simultaneously, acting as the only slow transduction pathway connecting the atria and ventricles electrically.

Histologically, the mid-nodal area is composed of cells that are densely distributed in a basket-like configuration; it extends between the His bundle and loose atrial pathways that lead to the node. The atrial and nodal cells are mainly composed of transitional cells; significant electrical and morphological specialization progressively occur in the distal His fibers.

### 2.2 His Bundle 

Wilhelm His Jr was the first to describe the His bundle in 1893. It is a specialized elongated structure that extends from the AVN and facilitates electrical conduction between the atria and ventricles [13]. The His bundle is located in the membranous part of the inter-ventricular septum (IVS) and can be divided into the penetrating, non-branching, and branching portions [11, 14]. The penetrating portion of the His bundle passes through the fibrous membranous septum or central fibrous body close to the mitral ring, and the septal portion runs a variable course along the crest of the muscular ventricular septum. The His bundle is a specialized cylindrical muscular structure that measures 2.6 mm (length) × 3.7 mm (width) × 1.4 mm (thickness). Histological analysis has shown collagenous septa that split the longitudinal strands of the bundle into distinct parallel compartments; the longitudinal dissociation theory is supported by the existence of these septa [15]. Pacing the distal His bundle can eliminate LBBB, which could potentially be explained by the septa [16]. Upon histological examination, the His bundle fibers also exhibit relatively few pacing or P cells. Notably, the left and right ventricles are activated virtually simultaneously due to the rapid transmission of electrical signals through the His–Purkinje system.

### 2.3 Bundle Branches

The His bundle subsequently branches into the left posterior fascicle, left anterior fascicle, and right bundle branch (RBB). The RBB extends from the right side of the IVS to the apex of the RV. It passes from the septum to its trabecular margin and reaches the base of the anterior papillary muscle. At this point, it separates and continues to form the subendocardial plexus of ventricular conduction cells or Purkinje fibers, which represent the final component of the cardiac conduction system. This network of specialized cells spreads throughout the ventricle and supplies ventricular muscle tissue, including the papillary muscles [17]. The left bundle branch (LBB) traverses under the endocardium on the left side of the septum and branches into the septal, anterior, and posterior fascicles [18]. As the His bundle and a part of its division cross the membranous septum, the length of the latter has an impact on the possibility of injury to the LBB and its branches during surgery for aortic valve pathologies [19]. The left anterior fascicle is thin and long, while the left posterior fascicle is broad and short [15]. The broader distribution facilitates LBBP, which is slightly easier to perform than HBP.

## 3. Definition, Categories, and Hemodynamic Effects of CSP

AVN pacing typically has a conduction time of 120–200 ms from pacing spike to ventricular capture, whereas His bundle pacing has a shorted time of 35–55 ms. A common aspect of all CSP sites is a very short overall peak time of left activation, which will result in a slightly varied QRS shape and clinical effects.

CSP can be classified as either selective or non-selective pacing based on the involvement of the local myocardium. In selective pacing, the surface electrocardiogram (ECG) shows a platform between the pacing signal and origin of the QRS complex, while in non-selective pacing, the QRS morphology is similar to that of pre-excitation syndromes. According to the location of the pacing lead, the current conduction bundle pacing can be roughly divided into AVN pacing [20], His bundle pacing, LBB/RBB pacing, and left anterior (posterior) branch pacing. CSP has many advantages and disadvantages; we have summarized them in Table 1 and discuss them in detail in the following sections.

**Table 1.** **Advantages and disadvantages of CSP**.

Type of CSP	Advantages	Disadvantages or limitations
HBP	(1) Narrows QRS duration compared with the traditional pacing pattern.	(1) Difficulties in mapping the His bundle area.
	(2) Reduces clinical events after implantation compared with traditional RVP methods.	(2) Capture of the conduction system needs a higher pacing threshold and can lead to early termination of battery life-expectancy.
	(3) Improves heart failure-related parameters more effectively than traditional methods.	(3) Improvement of prognosis needs long term observations.
	(4) Avoids the occurrence of functional tricuspid regurgitation, since atrial side HBP has no lead across the tricuspid valve.	(4) Atrial side HBP exhibits a higher pacing threshold.
LBBAP	(1) Lowers the pacing threshold compared to HBP.	(1) Lack of clinical evidence for guideline makers to provide recommendation levels.
	(2) More efficient in improving LBBB than HBP.	(2) Procedure involves fixation of leads to the depth of the septum; thus, perforation is likely.
	(3) Recovers the synchrony contraction of ventricles.	(3) During implantation, injury to the septum branch of the LAD will lead to the spasm of the LAD and acute coronary syndrome.
	(4) Since the LBB covers a large area of the left ventricle and the left side of the interventricular septum, the mapping is relatively easy, and the success rate is high.	(4) Lack of consensus criteria to identify LBB capture and distinguish LBBP from LVSP; the difference in clinical outcome between LVSP and LBBP is rarely studied.
	(5) Shortens the QT interval compared to RVP.	

CSP, conduction system pacing; RVP, right ventricular pacing; LBBAP, left bundle bunch area pacing; LBBB, left bundle branch block; HBP, His bundle pacing; LAD, left ascending artery; LVSP, left ventricular septal pacing; LBB, left bundle branch.

**Fig. 2.**
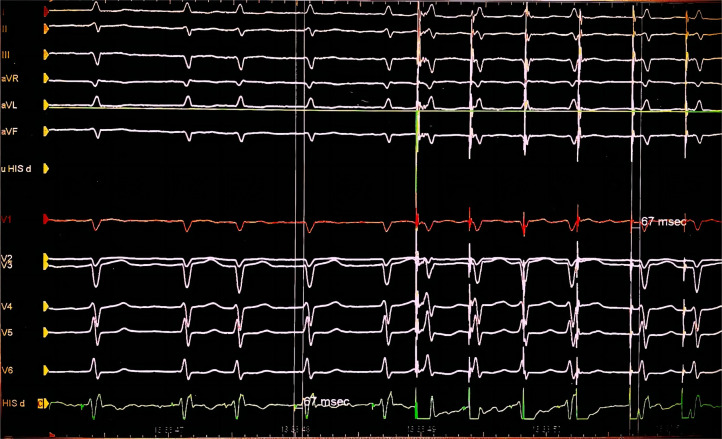
**Electrocardiography of successful His bundle pacing**. Successful His bundle pacing with an intrinsic QRS complex. The interval between the pacing spike and QRS onset (67 ms) is equal to that between the intrinsic His potential and QRS onset (67 ms).

**Fig. 3.**
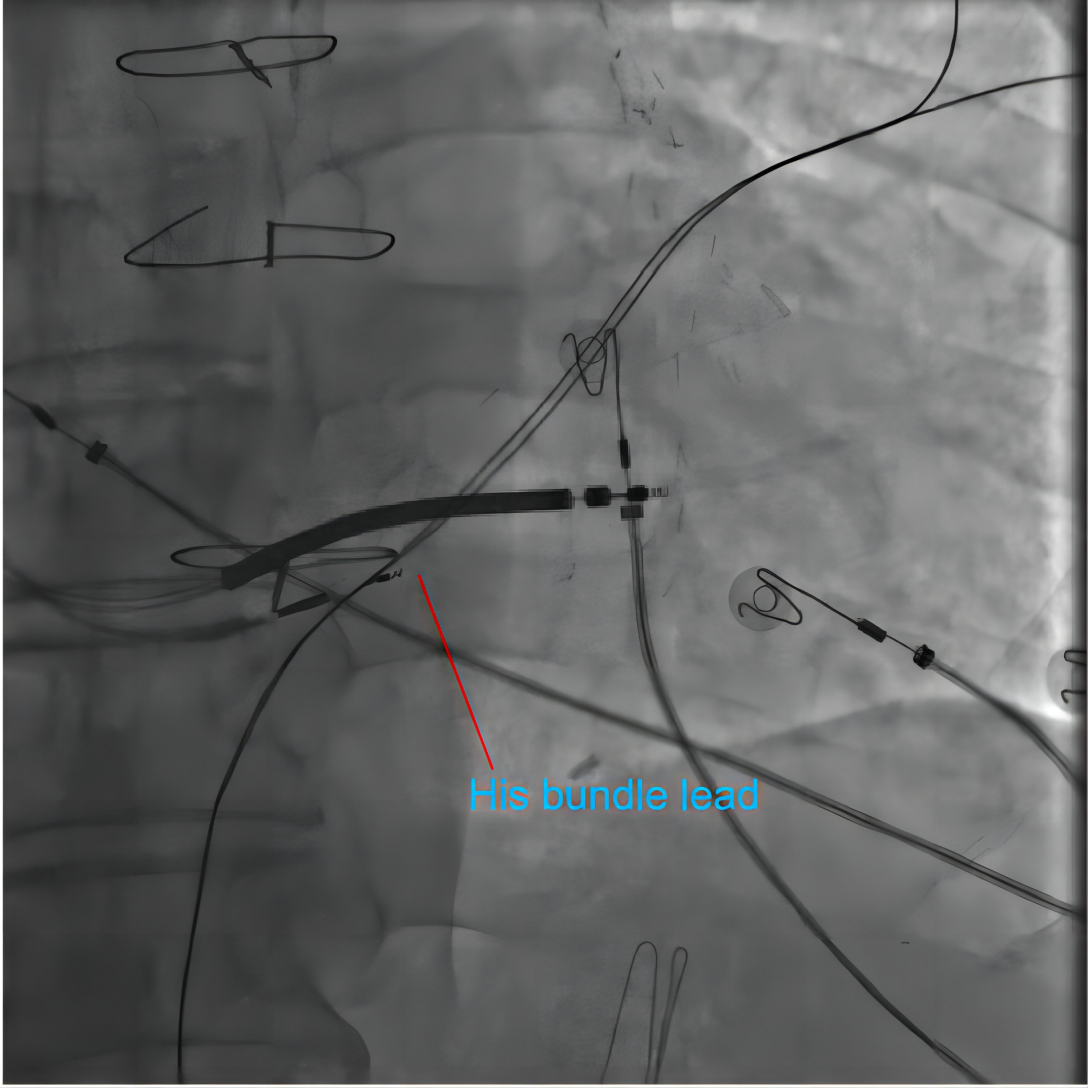
**Fluoroscopy of His bundle pacing**. In a patient receiving His bundle pacing-modified cardiac resynchronization therapy (CRT) therapy, the fluoroscopy image shows the position of the 3830 leads pointing in the His bundle area. The point of the 3830 leads is closer to the proximal side of the defibrillation lead. The defibrillation lead was placed in the lower septum.

HBP involves the direct capture of the AV bundle or His bundle. Consequently, simultaneous activation of all of the distal fibers is achieved [21]. Successful capture of the His bundle is initially determined by the following criteria: an intrinsic QRS complex, and the interval between the pacing spike and QRS onset equals that between the intrinsic His potential and QRS onset (as shown in Figs. 2,3), and His bundle capture demonstrates an all or none pattern, with the disappearance of the QRS wave in cases of low output [22]. Based on location and pacing outputs, HBP may be selective (isolated recruitment of the His bundle) or non-selective (recruitment of both the local septal myocardium and the His bundle).

**Fig. 4.**
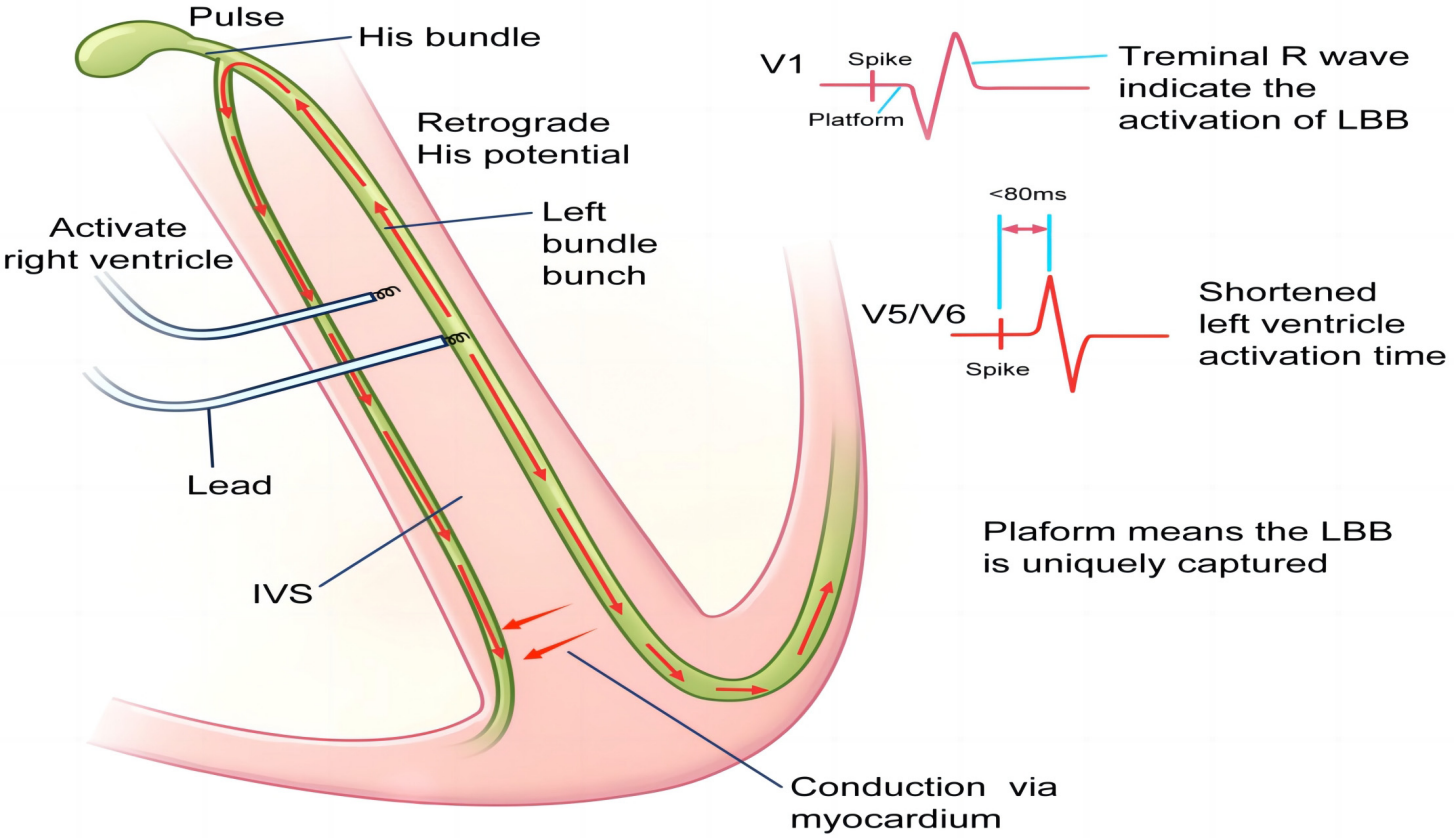
**Scheme of left bundle branch pacing (LBBP) mechanisms**. Scheme showing the mechanisms of electrocardiography and the electrophysiological characteristics of LBBP. The relative advanced pacing of the left ventricle by LBBP leads to the terminal R wave in the V1 lead and shortened R-wave peak time in the V5/V6 leads. Retrograde activation in the left bundle branch (LBB) causes retrograde His bundle activation and subsequent right bundle branch (RBB) activation. Subsequently, the RBB block can be rectified. Furthermore, the stimuli conducted via the conduction system can also transmit along the myocardium to activate the distal RBB. IVS, inter-ventricular septum.

**Fig. 5.**
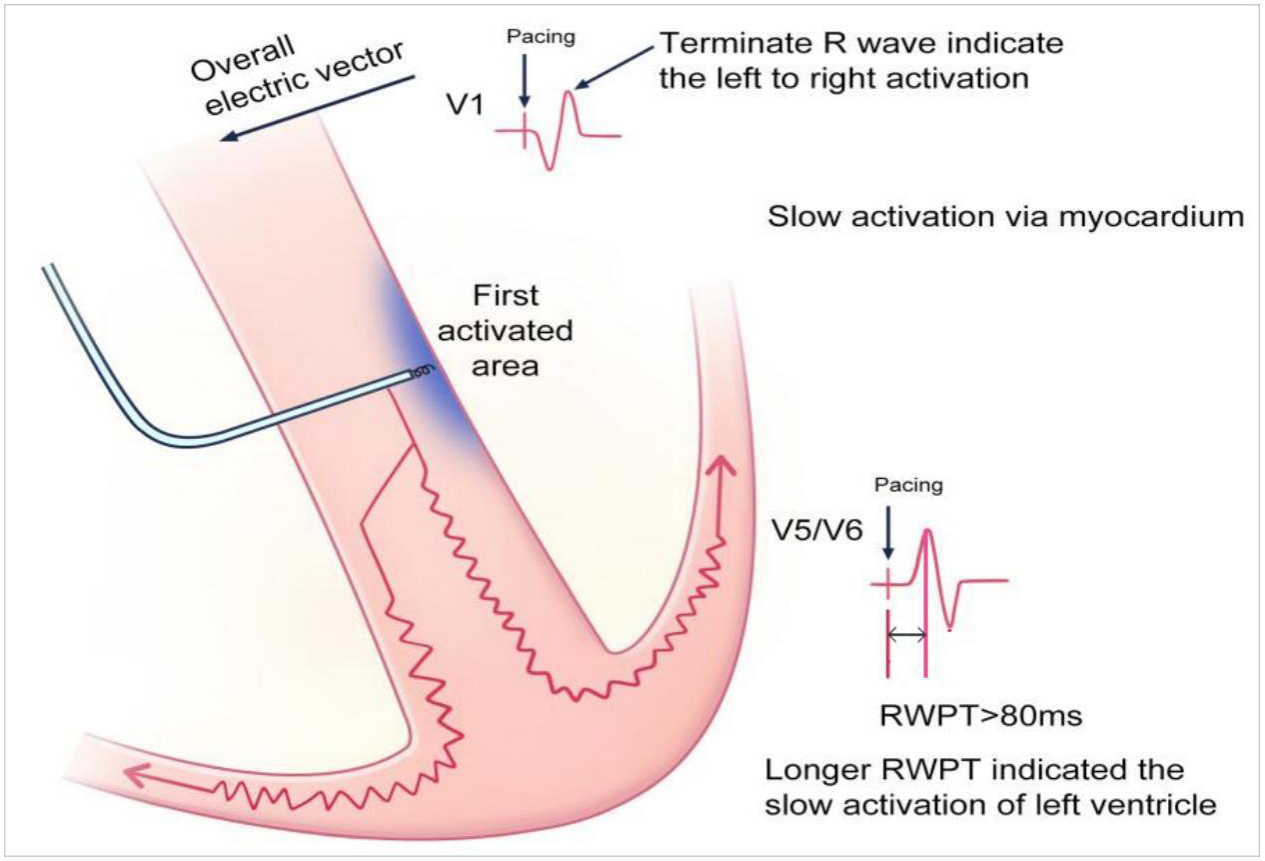
**Scheme of left ventricular septal pacing (LVSP) mechanisms**. Scheme showing the mechanisms of electrocardiography and electrophysiological characteristics of LVSP. LVSP involves activation of the left side of the septum. The stimuli are transmitted along the myocardium, and the activation is more lagging in left bundle branch block (LBBP). Thus, a longer R-wave peak time (>80 ms) was encountered. RWPT, R wave peak time.

LBBP is a type of ventricular pacing that is intended to stimulate the entirety or a portion of the LBB fascicular system. Similar to HBP, selective LBBP is defined as direct stimulation and isolated recruitment of the LBB fibers, and non-selective LBBP is defined as direct stimulation and recruitment of both the local myocardium and the LBB fibers (shown in Figs. 4,5) [23]. Both LBBP and left ventricular septal pacing (LVSP) are included in LBBAP. The general ECG manifestation of success LBBAP is the appearance of the RBB pattern in V1, LBB potential, shortened QRS duration, and a prolonged V6–V1 interpeak interval [24, 25, 26].

A previous study indicated that multipoint pacing (MPP) is an efficient way to improve non-invasive hemodynamic parameters such as the dP/dtmax (maximum rate of LV pressure elevation) and global longitudinal strain (GLS) [27]. Specifically, the dP/dtmax is a parameter used to measure the response of CRT. The underlying reason of improved hemodynamic parameters in MPP might be the simultaneous contraction provided by MPP [28]. Thus, MPP is an efficient modality for achieving CRT. CSP yields a more simultaneous contraction as it captures the specified conduction structure. The intrinsic conduction system is the fastest way to conduct electric signals in the heart. Thus, CSP is an ideal pattern of CRT. Current studies have indicated the advantages of CSP-modified CRT, improving the hemodynamic parameters compared to classic BiVP CRT, which is discussed in the following chapter.

## 4. Benefits and Challenges in HBP 

### 4.1 HBP and its Clinical Outcomes 

Deshmukh *et al*. [22] (2000) first revealed the feasibility of HBP; permanent HBP improved the LVEF and cardiac remodeling after 2 years of follow-up. The pacing location was either at the atrial or ventricular side [21]. HBP is superior to traditional pacing patterns in its improvements in clinical performance.

A recent meta-analysis proved that HBP produced a greater increment in the LVEF and reduced cardiac remodeling, all-cause mortality, and re-hospitalization rates than RVP and BiVP [29]. Notably, HBP is still beneficial in patients where BiVP failed, and the LVEF increased from 26 ± 9% to 41 ± 13% in patients with LBBB and from 32 ± 9% to 49 ± 11% in those without LBBB [30]. Vijayaraman *et al*. [31] found that after 5 years of follow-up, patients in the cohort who received RVP demonstrated a significantly higher risk of HF-related hospitalization or mortality than those who received HBP (41% vs. 27%, *p* = 0.04). These findings suggest that the clinical benefits of HBP need to be evaluated over a long time.

Individuals diagnosed with atrial fibrillation (AF) and HF may benefit from AVN ablation (AVNA) as a treatment for uncontrolled tachycardia [32]. HBP provides benefits over RVP in patients with AF receiving AVNA, and the ALTERNATIVE-AF trial demonstrated a higher improvement in the LVEF [33]. However, the increases in mortality, cardiovascular hospitalization, and LVEF did not reach statistical significance. This is likely because more patients switched from the BiVP arm to the His-CRT arm, which had a comparatively smaller sample size and lower baseline ejection fraction values [34]. The His-SYNC pilot trial, which included 41 patients, demonstrated that His-optimized CRT is superior to BiV-CRT in QRS duration and synchrony of ventricular electric activity [35]. In patients with LBBB, HBP also exhibited efficiency in improving the hemodynamic parameters compared with those who accept BiVP [8, 36]. Data demonstrating the advantages of HBP is presented in Table 2 (Ref. [8, 29, 30, 31, 33, 35, 36]).

**Table 2.** **Summary of the advantages of His bundle pacing strategies compared with traditional pacing**.

Author (year)	Study type	Control group	Selection criteria	Number	Advantage of HBP	Ref.
Paluszkiewicz P *et al*. (2024)	RCT	BiVP	Symptomatic heart failure, LVEF <35%, and LBBB	50 (1:1 allocated to both groups)	(1) Higher LVEF increment.	[8]
					(2) Lower end-systolic volume.	
Guan L *et al*. (2023)	MA	BiVP	Not available	1445	(1) Increased LVEF and decreased LVED.	[29]
					(2) Shorter QRS duration.	
Sharma P *et al*. (2018)	OS	BiVP	(1) Patients with HF	95	(1) Rescue failed BiVP by increasing LVEF.	[30]
			(2) Patients with failed BiVP or first attempt of HBP		HBP is a safe and feasible primary option for CRT in cardiomyopathy, AV block, LBBB or higher (>40%) anticipated pacing percentage patients.	
					(2) HBP improves QRS duration and New York Heart Association functional class.	
Vijayaraman P *et al*. (2018)	OS	RVP	Bradycardia	75 with HBP, 98 with RVP	(1) HBP reduced the combined endpoint risk of death or HF hospitalization during the 5-year follow-up.	[31]
					(2) In patients with >40% VP, HBP also showed the above advantage.	
Huang W *et al*. (2022)	RCT	BiVP	Persistent AF and reduced LVEF (≤40%) after AVN ablation	50	During the 18 months’ follow-up, HBP showed significant LVEF improvement.	[33]
Upadhyay G *et al*. (2019)	RCT	BiVP	Patients with indications for CRT	21 HBP and 20 BiVP-CRT	Significant reduction in QRS duration in the follow-up of 6.2 months.	[35]
Kato H *et al*. (2022)	CS	BiVP	Patients with LBBB and HF	14	Significant improvement in the dilation index (negative dP/dtmax).	[36]

RCT, randomized controlled trial; OS, observational study; MA, meta-analysis; CS, comparative study; LBBB, left bundle branch block; BiVP, biventricular pacing; HBP, His bundle pacing; AF, atrial fibrillation; LVEF, left ventricular ejection fraction; AVN, atrioventricular node; CRT, cardiac resynchronization therapy; HF, heart failure; VP, ventricular pacing; RVP, right ventricle pacing; AV, atrioventricle; LVED, left ventricle end-systolic diameter.

### 4.2 Challenges in HBP

Despite the numerous advantages of HBP, the high capture threshold is a vital challenge [37]. The unique anatomy of the His bundle allows for HBP located on the atrial side and hence, functional regurgitation is avoided [38]. However, it is associated with the issue of higher capture thresholds at baseline (*p* = 0.014) and at follow-up (*p* < 0.001) [39]. Usually, higher outputs are required to catch the His bundle, which may result from the fibrous tissue surrounding it. The capture thresholds are typically increased in less than a year.

The His bundle location and lead fixation are also challenging. HBP is not widely used in patients with larger ventricular canals because it is difficult to locate the His bundle [40]. It is always anticipated that the unique delivery sheath will facilitate the lead’s passage to the tricuspid annulus and speed up the localization of the His bundle [41]. His bundle visual mapping has been enhanced by recent advances in three-dimensional electro-anatomical mapping techniques (e.g., the KODEX-EPD system). In addition, the use of intra-cardiac echography (ICE) has eliminated the need for radiography during lead placement [42, 43]. A univariate analysis showed that non-U shaped slack is associated with the risk of threshold increase (*p* = 0.026), which implies that some threshold increase might be avoided [44]. The administration of anti-arrhythmic drugs, such as amiodarone, is related to a threshold increase [45].

### 4.3 Complications of HBP

Regarding the complications related to CSP, current large-scale data is rare [46]. A complication unique to HBP is an elevated pacing threshold during follow-up [47]. Surgical complications showed no significant difference between the HBP and RVP [48]. Device-related complications are more common in patients receiving HBP than those receiving RVP and LBBAP [49]. The occurrence of these complications is due to the narrow anatomical position of the His bundle, making it more challenging to target the right site. Furthermore, micro dislodgement of the lead is part of the reason for the elevated threshold [50]. However, with the development of surgical devices, such as the C315 His transmission sheath, the success rate of HBP can be guaranteed and complications can be reduced. During the follow-up, programming is also important, and a higher output voltage can avoid the loss of capture in patients undergoing HBP.

## 5. Benefits and Challenges in LBBAP 

### 5.1 LBBAP and its Clinical Outcomes

In 2017, Huang *et al*. [51] were the first to describe LBBP. They screwed the lead to the left side and corrected the LBBB because HBP was unable to do so. For individuals suffering from HF and LBBB, LBBAP is approved with a recommendation level of IIa according to the most recent Heart Rhythm Society guidelines [52].

LBBAP demonstrates advantages over traditional RVP and BiVP in terms of clinical outcomes. Numerous randomized controlled trials (RCTs) have shown that LBBAP offers better performance than RVP and BiVP in terms of mortality reduction, LVEF improvement, lowering N-terminal B-type natriuretic peptide levels, decreasing LV end-systolic volumes, and lower re-hospitalization rates [53, 54, 55]. In addition, LBBAP offers an advantage over RVP in reducing the occurrence of new-onset AF [56]. The above results were also proven in a meta-analysis [57]. These advantages have been summarized in Table 3 (Ref. [53, 54, 55, 56, 57]). In addition, the tip of the lead is enmeshed in the muscular component; therefore, ventricular lose capture is rare, enhancing the safety of pacing.

**Table 3.** **Summary of the advantages of LBBAP strategies compared with traditional pacing**.

Author (year)	Study type	Control group	Number of patients	Advantage of LBBAP	Ref
Sharma P *et al*. (2022)	OS	RVP	321 LBBAP and 382 RVP	(1) Lower risk of primary composite events (all-cause mortality, HFH, or upgrade to biventricular pacing).	[53]
				(2) Among patients with ventricular pacing burden >20%, the above advantages can be observed.	
Wang Y *et al*. (2022)	RCT	BiVP-CRT	40 consecutive patients	(1) Significant LVEF improvement (in the 6-month follow-up).	[54]
				(2) Greater reductions in left ventricular end-systolic volume.	
Liu Q *et al*. (2021)	RCT	RVP	84 patients (42 patients with RVP, 42 patients with LBBP)	(1) Lower levels of BNP.	[55]
				(2) Better left ventricle diastolic function 7 days after implantation.	
Zhu H *et al*. (2023)	PCS	RVP	257 LBBAP and 270 RVP	(1) During a mean follow-up of 11.1 months, LBBAP resulted in significantly lower incidence of new-onset AF.	[56]
				(2) In patients with higher VP (≥20%), LBBAP was associated with decreased risk of new-onset AF compared with RVP. However, the advantage of pacing modalities was not pronounced in patients with VP <20%.	
Jin C *et al*. (2023)	MA	BiVP	616 patients from 15 centers	(1) ECG and ultrasound parameters: LBBP has shorter QRS duration and higher LVEF improvement, and a greater LVEDD reduction.	[57]
				(2) Clinical outcomes: Improvement in New York Heart Association function class was significant in LBBAP. LBBAP lowers the risk of a composite of HFH and all-cause mortality.	

OS, observational study; RCT, randomized controlled trial; PCS, prospective cohort study; MA, meta-analysis, LBBAP, left bundle block atrial pacing; RVP, right ventricular pacing; BiVP, biventricular pacing; CRT, cardiac resynchronization therapy; HFH, heart failure hospitalization; LVEF, left ventricular ejection fraction; BNP, N-terminal B-type natriuretic peptide; AF, atrial fibrillation; VP, ventricular pacing; ECG, electrocardiography; LBBP, left bundle branch pacing; LVEDD, left ventricular end diastolic duration.

Conduction disturbances (high-degree AV block and new-onset LBBB) are common among patients who undergo trans-catheter aortic replacement using self-expanding valves [58]. LBBAP offers promise in the management of complications after trans-catheter aortic replacement. The hybridization of LBBAP with conventional pacing patterns has been demonstrated to yield significantly better electrical synchronization when compared to the use of either modality alone (LBBAP-optimized CRT) [59]. Ablation with LBBAP is an additional useful method for controlling heart rate in patients with AF who do not respond to medication therapy. The viability and safety of this hybrid method have been demonstrated by recent research [60]; however, the study cohorts are from single centers and the sample sizes are modest. To verify the benefits of this method, more thorough research is required.

In contrast to HBP, LBBAP is not linked to an elevated pacing threshold (resulting in reduced battery life). Nonetheless, few sizable clinical trials have examined the risks associated with surgery and assessed the safety of LBBAP. The procedure is associated with complications, as it involves screwing of the leads into the ventricular septum; the complications include septal perforation, thromboembolism, RBB injury, and lead dislodgement. Among them, septal perforation and thromboembolism are the most common. Approximately 3% of patients receiving LBBAP develop acute lead perforation [61], manifested by a decrease in R wave amplitude, increase in capture threshold, or an immediate decline in unipolar impedance to below 500 Ω [62]. In 2020, Ponnusamy and Vijayaraman [63] reported a rare case of myocardial infarction, which was caused by arterial spasms induced by the LBBAP implantation process. As the LAD artery gives rise to the septal branch, screwing of the lead in the septum may damage the left ascending artery (LAD) and lead to ST-elevation myocardial infarction [63].

### 5.2 Optimal Criteria for Left Conduction System Capture

Ideal LBBP captures the trunk of the LBB prior to branching, and the fascicles arising from the LBB trunk are activated simultaneously. Left fascicular branches extend from the main trunk of the LBB and terminate before the Purkinje fiber network, and more than half of the LBB-related pacing site is located in the left fascicular area [64]. Selective LBBP exhibits isoelectric intervals between pacing spikes and QRS complexes [65].

LBBAP demonstrates intra-ventricular electrical synchrony as opposed to inter-ventricular electrical synchrony, which produces the terminal R’ wave seen in lead V1. Retrograde conduction from the left bundle to the right bundle, myocardial septal capture, or fusion with native right bundle conduction, on the other hand, may eliminate delayed RV conduction. Insufficient depth pacing typically displays a deep septal pacing pattern (DSP) and is unable to capture fascicles or the main stem of the LBB. In contrast to LBBAP, DSP does not illustrate the characteristics of CSP although the QRS complex might not be obviously prolonged [66].

LBBAP functions by generating two conductions, and the retrograde HB potential and anterograde distal LBB potential might be recorded. LBB potential is a sharp high-frequency deflection 15–30 ms before the onset of surface QRS [67]. Certain criteria have been used to differentiate between LSVP and LBBAP. Theoretically, there are direct proofs for LBB capture provided by the LBB potentials, retrograde His bundle capture, or early distal anterograde left conduction system potentials during LBBAP [68]. However, these criteria have certain limitations. The septal myocardial refractory period is shorter than the LBB refractory period, which makes programmed deep septal stimulation useful for confirming LBB capture. LBBAP may be categorized as LBBP, left fascicular branch pacing (LFBP), and LV septal pacing (LVSP). Current criterion to differentiate LBBP from LVSP is summarized in Table 4 (Ref. [24, 25, 26, 64, 67, 68, 69, 70]).

**Table 4.** **Criterion to differentiate LBBP from LVSP**.

Criteria for LBB capture	Criteria to identify LVSP
**QRS complex:**	(1) LVSP was defined if the only fulfilling criterion was that of paced QRS morphology in lead V1 demonstrating QR or QS pattern [24, 26].
(1) W-shaped morphology in V1 and QRSd <145 ms [68, 69].	(2) Fluoroscopic confirmation of the pacing lead position in basal/mid-septal region was needed to exclude the presence of characteristic QRS morphology in V1 due to apical lead position [64].
(2) Alternation of QRS morphology after lead screwing in the septum [68, 69].	(3) QR or QS pattern in lead V1, R wave without any notch in lead V6, and none of the above LBBP criteria were met [68].
(3) Narrow-paced QRSd in RBBB pattern <130 ms after screwing in the LBB area [69].	
(4) Transition from non-selective LBB capture to selective LBB or LVSP only capture observed during decreasing output [24, 26, 64, 68, 69].	
**Interval between the pacing spike and QRS complex:**	
(1) Stimuli to QRS interval is equal to or longer than the LBB potential to QRS [69].	
(2) Abrupt shortening of the interval from pacing spike to the peak of R wave in V5 or V6 (S‐LVAT) >10 ms was observed while changing output, or the difference between S‐LVAT and P_L⁢B⁢B_-QRS <5 ms [24, 26, 64].	
(3) Stimuli to QRS interval is equal to or longer than the LBB potential to QRS [69].	
(4) Pacing stimulus to V6RWPT <80 ms in patients with narrow QRS/isolated RBBB or <90 ms in patients with more advanced ventricular conduction system disease [64].	
**LVAT/RWPT:**	
(1) ΔLVAT1 (LVAT_H⁢B⁢P_ – LVAT_L⁢B⁢B⁢P/L⁢V⁢S⁢P_) >12.5 ms in non-HF patients and ΔLVAT1 (LVAT_H⁢B⁢P_ – LVAT_L⁢B⁢B⁢P/L⁢V⁢S⁢P_) >9.0 ms in HF patients; ΔLVAT1% ((LVAT_H⁢B⁢P_ – LVAT_L⁢B⁢B⁢P/L⁢V⁢S⁢P_)/LVAT_H⁢B⁢P_) >14.8% in non-HF patients and ΔLVAT1% ((LVAT_H⁢B⁢P_ – LVAT_L⁢B⁢B⁢P/L⁢V⁢S⁢P_)/LVAT_H⁢B⁢P_) >9.8% in HF patients [26].	
(2) Absolute value of LVAT <75.0 ms showed a good predictive value for LBBP in non-HF patients and LVAT <85.0 ms showed a good predictive value for LBBP in HF patients [26].	
(3) Abrupt shortening of RWPT (≥10 ms) during lead implantation. Once the LBB is captured, RWPT remains constant across different pacing outputs [67].	
**V6**–**V1 interpeak interval:**	
(1) V6–V1 interpeak interval >40 ms [64].	
(2) V6–V1 interpeak interval >33 ms favors LBB (ns-LBB) capture [25, 68].	
**Other criteria:**	
(1) Tip of the pacing lead was directly against the septum and confirmed in the left anterior oblique 45 position [69].	
(2) Stimulus to Phis = intrinsic His potential – PLBB (Stimulus His: <35 ms) [70].	
(3) Demonstration of anterograde left conduction potential preceding the ventricular EGM on the multielectrode catheter during LBBP from the LBBP lead when there is intact stimulation to left conduction system [70].	
(4) Retrograde activation of the His bundle [67].	

LBBP, left bundle branch pacing; LVSP, left ventricular septal pacing; QRSd, QRS duration; RBBB, right bundle branch block; LBB, left bundle branch; RWPT, R wave peak time; HF, heart failure; LVAT, left ventricular activation time; S-LVAT, stimuli to the activation of left ventricle; P_LBB_, potential of left bundle bunch; HBP, His bundle pacing; ns-LBB, non selective left bundle bunch pacing; EGM, electrogram.

### 5.3 Challenges in the LBBP Procedure

The His bundle is a landmark for LBBAP, and pace-mapping is also required (V1 displays a “W” morphology with a mid-notch) to locate the LBBAP area. Sometimes the nine-partition method is helpful to localize the region for successful LBBAP [71]. More recently, Liu *et al*. [72] described a contrast-based visualization technique by defining the tricuspid valve in 60 patients undergoing LBBP.

**Fig. 6.**
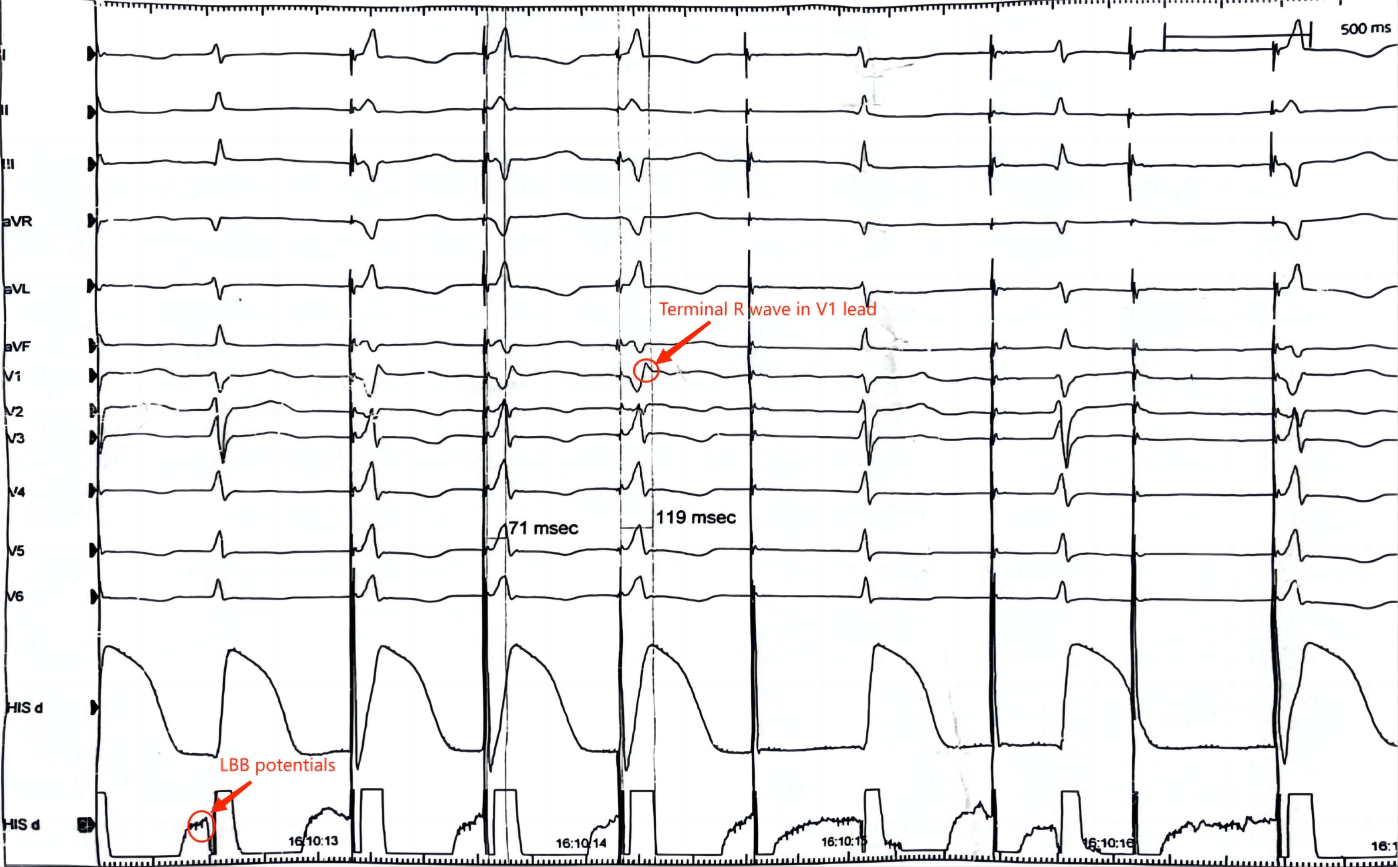
**Electrocardiogram of a patient receiving left bundle branch atrial pacing (LBBAP)**. In a successful LBBAP procedure, the LBB potential (a high frequency and low amplitude fragment spikes ahead of the QRS wave) can be seen in the His lead, and after pacing at 2 V, the terminate R wave can be seen. LBB, left bundle branch.

**Fig. 7.**
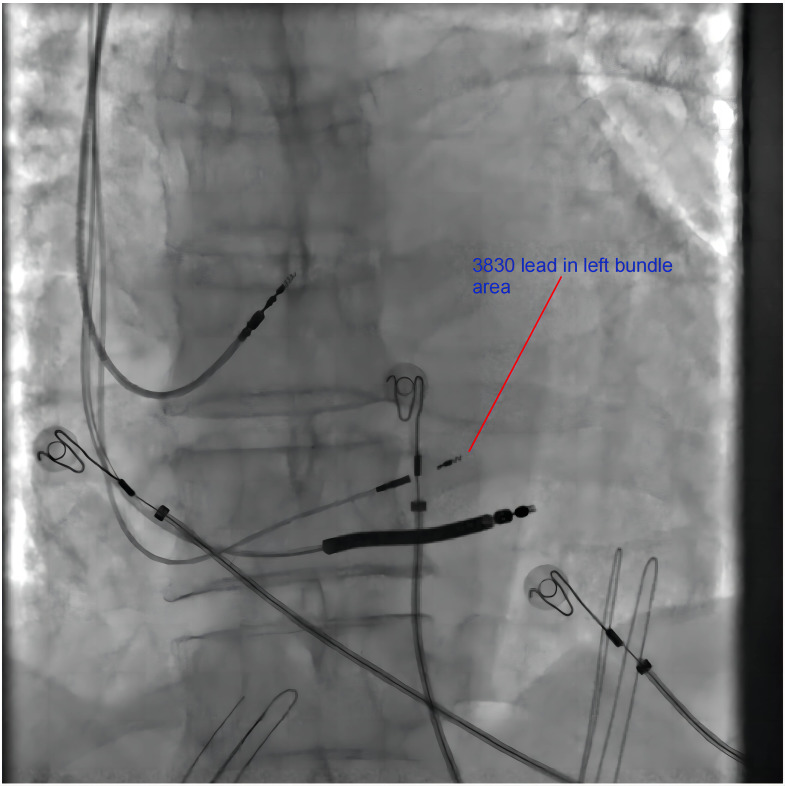
**Fluoroscopy of a patient receiving left bundle branch atrial pacing (LBBAP)-modified cardiac resynchronization therapy**. The fluoroscopy image of the position of the 3830 leads pointing in the LBB area. The 3830 lead is closer to the distal end of the defibrillation lead. LBB, left bundle branch

Continuous fluoroscopy is one of the methods used for monitoring insertion depth (Fig. 5). A technique for depth monitoring is also provided by evaluation of the ECG during unipolar pacing. To determine LBB capture, simultaneous monitoring of the peak left ventricular activation time (LVAT) in leads V5 or V6 is helpful. The LVAT shortening and the emergence of an LBB potential are indicative of LBB capture (Figs. 6,7). The fixation (template) beats are also useful to identify the depth of insertion. Myocardial current of injury (COI) is also an indicator of lead depth; a high COI suggests further insertion of the lead to be safe. A clear drop in the COI amplitude (to approximately 5 mV) and the appearance of Purkinje potentials indicate the need for caution during further screwing of the lead. In real-world clinical situations, the mapping of His bundle potentials may be omitted. Direct monitoring of the LVAT and changes in QRS morphology in lead V1 are also helpful indicators.

The KODEX mapping system employs new electrophysiological anatomical mapping and navigation technology, and it can provide a three-dimensional view of the heart chambers during radiofrequency ablation and may help map the location of the His bundle and 3830 lead [73]. Kuang *et al*. [74] used the CARTO and ICE systems to map the conduction system (including the LBB and His bundle); they subsequently used ICE to guide LBB localization and monitor lead insertion into the septum. This demonstrates the advantages offered by these newer technologies over traditional methods in terms of procedure intervals and fluoroscopic exposure times [74].

In cases where IVS penetration is difficult, the sheath should be checked to ensure correct orientation. Sometimes longer and more supportable sheaths are required in stuff cases. The sheath may be repositioned 1–2 cm away from the LBB truck area to avoid the scar tissue. Pulling the sheath back slightly after anchoring the leads with several rotations (while maintaining counterclockwise torque to provide good contact and support force) will straighten the proximal curve and facilitate septal penetration.

## 6. Clinical Outcomes of Different CSP Modalities 

### 6.1 LBBP Compared to HBP for CRT

RV activation delay via LBBP could not be omitted, although LBBAP offers several technical advantages compared to HBP. Ali *et al*. [75] compared the acute hemodynamic results between HBP and LBBP to demonstrate whether the delayed RV activation decreased cardiac function. Non-invasive electrical mapping proved that HBP produces significantly more rapid biventricular activation than LBBAP (*p* = 0.03), although LBBAP was not inferior to HBP in LVAT (*p* = 0.65) and the delayed RV activation with LBBAP did not worsen the acute hemodynamic response obviously in terms of systolic pressure (*p* = 0.8) [75].

A recent study involving 121 patients with RBBB (107 patients having their RBBB) found that correction of the RBBB conduction disruption can increase the LVEF [76]. Another study showed that anodal capture can advance RV activation and result in decreased total ventricular activation times (*p* = 0.01), although the exact underlying mechanism remains unclear. Higher outputs are necessary, and hemodynamic differences are less significant in that study [77].

Moreover, a recent network meta-analysis involving 4386 patients from 33 studies compared the advantages among LBBAP, HBP, and BiVP. This study indicated that LBBP showed statistically significant advantages over HBP and BiVP in terms of LVEF, QRS duration reduction, rehospitalization rates, and lead issues [78]. Therefore, this non-head-to-head study provides evidence for LBBP being superior to other CSP modalities, including HBP.

### 6.2 LVSP Compared to LBBP for CRT

LBBP and LVSP demonstrate considerably different hemodynamic and electrophysiological characteristics [79]. Zhou *et al*. [80] found that the procedure time was significantly increased in LBBP compared with LVSP in patients with atrioventricular block. The LVAT was significantly shorter in LBBP than LVSP (*p* = 0.032). After 12 months’ follow-up, no significant decrease in cardiac function was detected in all patients [80]. However, Zhang *et al*. [81] found that LV mechanical synchrony during LBBP was significantly shorter than those during LVSP (*p* < 0.05). After 17 months’ follow-up, the improvement of LVEF in the LBBP was more obvious than that in LVSP (*p* = 0.01) [81]. However, the effect of such differences on clinical outcomes is not clear given the lack of studies on the clinical outcomes.

### 6.3 Developments in CSP Delivery Systems

With or without the Selectra 3D transmission sheath (Biotronik SE & Co. KG, Berlin, Germany), stylet-driven pacing leads (SDLs) can be effectively utilized for LBBAP by offering support force [82, 83]. Patients with a big heart cavity require a stronger support force for lead fixation. The safety and viability of utilizing SDLs for LBBAP have been shown by recent comparative studies and case reports (Table 5, Ref. [83, 84, 85, 86]) [84, 85, 86]; however, regulators have not approved the use of these leads in CSP.

**Table 5.** **Summary of feasibility comparison among different brands of leads in CSP practice**.

Author (year)	Objects	Number of patients	Pacing pattern	Outcome	Ref
Sun Y *et al*. (2022)	SDL (FINELINE II 4471 lead, Boston Scientific, Marlborough, MA, US) vs. LLL (Select Secure 3830 lead, Medtronic, Minneapolis, MN, US)	25 patients in the SDL group and 20 patients in the LLL group	LBBAP	Paced QRS duration and the stimulus to peak LVAT showed no statistically significance difference between the SDL and LLL groups.	[83]
				Pacing threshold and R-wave amplitude at baseline and follow-up showed no statistically significance difference between the SDL and LLL groups.	
De Pooter J *et al*. (2021)	SDL (Solia S60, Biotronik, SE & CO, Lake Oswego, OR, USA) and new delivery sheath (Selectra 3D, Biotronik, Lake Oswego, OR, USA) vs. LLL (Select Secure 3830 lead, Medtronic, Minneapolis, MN, USA)	23 patients in the SDL group and 27 patients in the LLL group	LBBAP	Success rate: 20/23 (87%) patients in the SDL group and 24/27 (89%) patients in the LLL group (*p* = 0.834). No difference was noted between the two groups.	[84]
				LBBAP thresholds and R amplitude showed no statistically significant difference between SDL and LLL at baseline and follow-up.	
				Post procedural echocardiography revealed a septal coronary artery fistula in one patient with SDL LBBAP.	
Sritharan A *et al*. (2023)	SDL from four manufacturers: Solia S 60 (Biotronik, Berlin, Germany), Tendril STS (Abbott, Sylmar, CA, USA), Ingevity (Boston Scientific, Marlborough, MA, USA), and Vega (Microport, Shanghai, China) delivered using the Selectra 3D 55 or 65 curve (39 or 42 cm catheter length) delivery system vs. 3830-69 SelectSecure lead (Medtronic, Minnesota, Minneapolis, MN, USA) delivered using the C-315 His sheath (or a Selectra 3D sheath if previously used unsuccessfully with an SDL)	153 patients in the SDL group and 153 patients in the LLL group	LBBAP	There was no difference in the success rate between the initial use of lead type (96.0% with SDL vs. 94.3% with LLL, *p* = 0.56). There were no significant differences in success among lead models.	[85]
				V6RWPT, QRS duration, conduction system capture, sensing amplitude, and pacing threshold showed no statistically significant differences between the SDL and LLL groups at baseline and follow-up.	
				Operation complications are similar between the two groups.	
Michael V Orlov *et al*. (2019)	Boston Scientific (Marlborough, MA, USA) 4471 and model 8107 (Boston Scientific) Acuity Pro sheath and 7742 SDLs vs. LLL (Select Secure 3830 lead, Medtronic, Minneapolis, MN, USA)	27 patients in the SDL group and 17 patients in the LLL group	HBP	HBP with SDL was successful in 24 of 27 patients (89%) compared to 15 of 17 patients (88%) in the LLL group.	[86]
				Acute and intermediate (4.7 ± 1.8 months) follow-up HBP thresholds were higher with SDL than with LLL, but chronic (long-term) pacing thresholds were stable in each group.	

SDL, stylet-driven pacing leads; LLL, lumen-less lead; RWPT, R wave peak time; LBBAP, left bundle branch atrial pacing; HBP, His bundle pacing; LVAT, left ventricular activation time; CSP, conduction system pacing.

Regarding the procedural devices (Table 6), newly developed SDLs, such as Solia S (Biotronik, SE & Co., Lake Oswego, OR, USA) and FINELINE II 4471 (Boston Scientific, USA), have been reported to be safe and useful [83, 84]. Transmission catheters for SDLs are also available (including the Abbott Agilis HisPro Catheter and Selectra 3D); they have a sturdier structure and offer strong support force. However, the indications for their use and the corresponding transmission catheters warrant further investigation, as the 3830 lead and 315 His sheath are used as standard for LBBAP [87].

**Table 6.** **Summary of the devices used for LBB procedures**.

Brand		Type of device	Approval status by FDA
Biotronik			
	Solia S	Stylet-driven pacing leads	Investigational device exemption is undergoing
	Selectra 3D catheter	Transmission catheter	Investigational device exemption is undergoing, but approved for HBP and LBBAP by Conformité Européenne Medical Device Regulation
Abbott			
	Agilis HisPro	Transmission catheter	Not applicable
Medtronic			
	3830 Pacing Lead	Lumen-less lead	Approved for HBP and LBBAP
	C315 Delivery System	Transmission catheter	Cleared for various lead implants; often used with HBP and LBBAP
Boston Scientific			
	FINELINE II 4471 lead	Stylet-driven pacing leads	Not applicable

LBB, left bundle branch; LBBAP left bundle branch atrial pacing; HBP, His bundle pacing; FDA, food and drug association.

### 6.4 Management of the Implanted Lead Wire in CSP

The adoption of CSP is anticipated to increase significantly in the coming years subject to further understanding of the benefits of this technique. Infection, malfunctioning, or redundant CSP leads are issues that need tackling [88]. Thus, the extraction of the lead wire is necessary in such situations. With the development of surgical devices, transvenous lead extraction (TLE) has more of a priority than in previous open-heart surgery. Recent clinical research has indicated that transvenous extraction of the commonly used 3830 lead in patients treated with CSP (both LBBAP and HBP) showed a high success rate (94%) and no major complications [89]. Only one lead in LBBAP was not fully extracted because the 3830 lead was drilled deep into the septum. Furthermore, in patients receiving chronic (25 ± 18 months) His bundle lead implantation, the extraction of the HBP lead had a high success rate (100% success in lead implantation ≤12 months and 95% success in lead implantation >12 months) and a low complication rate [90]. The above results indicate the safety and feasibility of HBP lead wire extraction. However, this does not mean that extraction of the LBB lead is unsafe. Previous studies have also indicated the possibility of LBBAP lead extraction without using instruments [91, 92]. One study reported a case of LBBAP lead extraction via the femoral vein pathway by using a double-loop design snare without any post-surgical complications [93]. This evidence indicates the convenience of LBBAP lead extraction.

### 6.5 Future Perspectives of CSP

Functional mitral regurgitation (MR) may result from desynchrony in the LV. CRT was demonstrated to reduce the severity of MR [94]; however, current studies rarely focus on the effect of CSP in reducing MR and, therefore, further investigation is needed. Theoretically, CSP is better at synchronizing the motion of the ventricle than the traditional BiVP modality. Thus, CSP is a promising approach to solve functional MR. This review also reveals the potential of HBP and LBBP in reducing the severity of tricuspid valve regurgitation, indicating the potential of CSP in treating valvular diseases [95].

As a new pattern of CSP, distal HBP has the potential to be an effective alternative to CRT [96]. According to the longitudinal dissociation theory, patients with LBBB may have lesions within the His bundle and only capturing the His bundle in the proximal part cannot cross the block site. Thus, targeting the distal part (or left side of the His bundle) is reasonable. Drilling deep into the left side of the septum is needed to penetrate the membranous part of the IVS to reach the distal part of the His bundle. Case reports have indicated that the distal HBP showed a superior local ventricular threshold during follow-up, safety, and feasibility in patients with a complex heart anatomy [97, 98]; however, currently no large-scale clinical trials have investigated the clinical outcomes of distal HBP.

The integration of leadless pacemakers (LLPM) with the experience of CSP is an attractive approach in terms of avoiding the complications associated with traditional pacemakers. With this purpose, the Wise-CRT system was devised to perform LBBAP with the LLPM. Contrary to traditional pacemakers, LLPMs are implanted in the LV. Current studies have indicated the feasibility and safety of LLPM in LBBAP [99, 100].

## 7. Conclusions

CSP is a promising method and superior approach to conventional pacing modalities in providing effective electromechanical ventricular synchronization. The pacing threshold for LBBAP is lower and the learning curve is shorter than that for HBP. The choices and outcomes of different CSP modalities may vary depending on the patient population. There are still several unanswered and unclear aspects concerning the efficacy of CSP. Thus, more large-scale prospective RCTs and more innovative solutions for the clinical procedures are required. We believe that CSP will have a more versatile application in fields other than arrhythmia.

## Abbreviations

AF, Atrial fibrillation; BiVP, Biventricular pacing; COI, Current of injury; CRT, Cardiac resynchronization therapy; CSP, Conduction system pacing; DSP, Deep septal pacing pattern; HBP, His bundle pacing; HF, Heart failure; HFH, Heart failure hospitalization; ICE, Intra-cardiac echography; IVS, Inter-ventricular septum; LBB, Left bundle branch; LBBAP, Left bundle branch area pacing; LBBB, Left bundle branch block; LBBP, Left bundle branch pacing; LFBP, Left fascicular branch pacing; LV, Left ventricular; LVAT, Left ventricular activation time; LVEF, Left ventricular ejection fraction; LVSP, Left ventricular septal pacing; MR, Mitral regurgitation; RA, Right apex; RBB, Right bundle branch; RCT, Randomized controlled trial; RVP, Right ventricular pacing; SDL, Stylet-driven pacing leads; TLE, Transvenous lead extraction.
